# Cocaine-induced plasticity, motivation, and cue responsivity do not differ in obesity-prone vs obesity-resistant rats; implications for food addiction

**DOI:** 10.1007/s00213-023-06327-5

**Published:** 2023-02-18

**Authors:** Anish A. Saraswat, Lauren G. Longyear, Alex B. Kawa, Carrie R. Ferrario

**Affiliations:** 1 Department of Pharmacology, University of Michigan, Ann Arbor, MI 48109 USA; 2 Psychology Department (Biopsychology), University of Michigan, Ann Arbor, MI 48109 USA; 3 Department of Behavioral Neuroscience, Oregon Health & Science University, Portland, OR 97239 USA

**Keywords:** Motivation, Behavioral economics, Self-administration, Intermittent access, Obesity

## Abstract

**Rationale:**

Compared to obesity-resistant rats, obesity-prone rats consume more food, work harder to obtain food, show greater motivational responses to food-cues, and show greater striatal plasticity in response to eating sugary/fatty foods. Therefore, it is possible that obesity-prone rats may also be more sensitive to the motivational properties of cocaine and cocaine-paired cues, and to plasticity induced by cocaine.

**Objective:**

To examine baseline differences in motivation for cocaine and effects of intermittent access (IntA) cocaine self-administration on cocaine motivation, neurobehavioral responsivity to cocaine-paired cues, and locomotor sensitization in male obesity-prone vs obesity-resistant rats.

**Methods:**

Intravenous cocaine self-administration was used to examine drug-taking and drug-seeking in males. Motivation for cocaine was measured using a within session threshold procedure. Cue-induced c-Fos expression in mesocorticolimbic regions was measured.

**Results:**

Drug-taking and drug-seeking, cue-induced c-Fos, locomotor sensitization, and preferred level of cocaine consumption (*Q*_0_) were similar between obesity-prone and obesity-resistant groups. Maximal responding during demand testing (*R*_max_) was lower in obesity-prone rats. IntA experience enhanced motivation for cocaine (*P*_max_) in obesity-prone rats.

**Conclusions:**

The results do not support robust inherent differences in motivation for cocaine, cue-induced cocaine seeking, or neurobehavioral plasticity induced by IntA in obesity-prone vs obesity-resistant rats. This contrasts with previously established differences seen for food and food cues in these populations and shows that inherent enhancements in motivation for food and food-paired cues do not necessarily transfer to drugs and drug-paired cues.

**Supplementary Information:**

The online version contains supplementary material available at 10.1007/s00213-023-06327-5.

## Introduction

The motivation to seek out and consume potentially addictive substances like cocaine relies on activity of mesocorticolimbic circuits that evolved to direct behavior towards essential reinforcers like food. Thus, it is not surprising that foods, addictive drugs and cues associated with these reinforcers share the ability to enhance activity within mesolimbic regions, including the nucleus accumbens (NAc; Tang et al. 2012). In addition, both food- and drug-seeking behaviors are powerfully influenced by cues associated with them. For example, food cues can elicit approach, spur on food-seeking behavior, and support instrumental responding in and of themselves; the same is true of drug-paired cues (Parkinson et al. 2000; Cardinal et al. 2002; Robinson and Flagel 2009; Derman et al. 2020; Robinson et al. 2015).

Cue-triggered urges to seek out food are often described as an adaptive component of successfully identifying and finding food in the environment, whereas urges triggered by drug-paired cues are often considered inherently aberrant; driving relapse and continued consumption that characterize drug addiction (Kawa et al. 2019a, b; Piazza and Deroche-Gamonet 2013). However, cue-triggered urges to seek out and consume calorie dense foods are stronger in people with obesity, occur in the absence of explicit caloric need, and predict future difficulty maintaining successful weight loss (Martin et al. 2010; Yokum et al. 2011; Murdaugh et al. 2012; Boswell and Kober 2016). This has led to complex and challenging questions about the degree to which neural and behavioral concepts of drug addiction can, or should, be applied to food and common obesity (Dagher 2009; Volkow et al. 2013; Ferrario 2017; Vainik et al. 2020).

Not all individuals are equally susceptible to weight gain and obesity (Albuquerque et al. 2015; Loos and Yeo 2022). This resulted in the development of rodent models that capture susceptibility and resistance to diet-induced obesity, including selectively bred obesity-prone and obesity-resistant rat lines (Giles et al. 2016; Levin et al. 1997; Alonso-Caraballo et al. 2018). Obesity-prone rats eat more than their obesity-resistant counterparts, and work more for food when the cost to obtain food is relatively low (Vollbrecht et al. 2015). In addition, prior to the development of obesity, motivational responses to food cues are stronger in obesity-prone compared to obesity-resistant rats (see Ferrario 2020 for review). Specifically, they show greater Pavlovian conditioned approach, stronger sign- and goal-tracking behavior, and enhanced Pavlovian-to-instrumental transfer in response to food cues compared to obesity-resistant rats (Robinson et al. 2015; Derman and Ferrario 2018; Alonso-Caraballo and Ferrario 2019). This is associated with increased intrinsic excitability of principle neurons in the NA﻿c in obesity-prone vs obesity-resistant rats that reduces the firing threshold of these neurons (Oginsky et al. 2015; Alonso-Caraballo and Ferrario 2019). Furthermore, obesity-prone rats also show enhancements in experience-induced plasticity within the NAc compared to obesity-resistant rats. For example, eating sugary, fatty “junk-foods” leads to persistent increases in calcium-permeable AMPA receptor (CP-AMPAR) transmission and expression within the NAc of obesity-prone but not -resistant rats (Oginsky and Ferrario 2019; Alonso-Caraballo et al. 2021; Nieto et al. 2022), and activity of these receptors has been tightly linked to cue-induced food- and cocaine-seeking behaviors (Crombag et al. 2008; Loweth et al. 2014; Derman and Ferrario 2018).

The pattern of behavior described above, and the overlap between motivational processes governing food- and drug-seeking, suggest that obesity-prone rats may also be more sensitive to the motivational properties of cocaine and cocaine-paired cues, and to plasticity induced by cocaine self-administration. To address these possibilities, we used intravenous cocaine self-administration procedures to examine drug-taking and drug-seeking behavior, and motivation for cocaine before and after intermittent access (IntA) self-administration experience in obesity-prone and obesity-resistant male rats. An IntA procedure was used because it has been shown to be particularly good at inducing addiction-like behaviors and associated neuroplasticity (Algallal et al. 2020; Allain et al. 2021; Calipari et al. 2013, 2014, 2015; Carr et al. 2020; Samaha et al. 2021; Kawa et al. 2016; Kawa and Robinson 2019; Kawa et al. 2019a, b). Motivation in the current study was measured using a within session threshold procedure that captures demand for cocaine (i.e., how consumption changes as a function of price) and the preferred level of consumption when price is negligible (Bentzley et al. 2013). In addition, we examined cue-induced c-Fos protein expression in the medial prefrontal cortex, amygdala, and NAc after IntA, and the induction of locomotor sensitization across IntA in obesity-prone and obesity-resistant groups.

## Methods

**Fig. 1:**
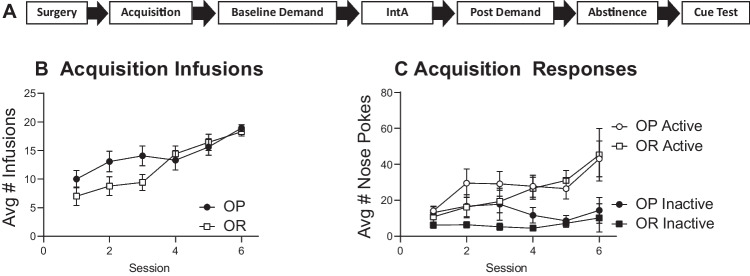
Experiment 1; acquisition of cocaine self-administration is similar across groups. **A** Experimental timeline. **B** Average number of infusions across each session in obesity-prone (OP, *n* = 12) and obesity-resistant (OR, *n* = 14) groups. **C** Average active and inactive responding across each session. All data shown as mean ± SEM

General methods are given first, followed by details of each experiment including Ns for each group. See also timelines shown in panel A of Figs. 1, 5, and 7.

### Subjects

Male selectively bred obesity-prone (OP) and obesity-resistant (OR) rats, originally developed by Barry Levin from outbred Sprague Dawley rats were used for all studies (OP *n* = 36, OR *n* = 39; Levin et al. 1997; see also Bouret et al. 2015, section VI, C2 and Ferrario 2020 for additional information about this model). Rats were bred in house (colonies maintained by the UM Breeding Core), and were group housed on a reverse light–dark cycle (12 h lights off at 08:00/12 h lights off at 20:00) in a climate-controlled colony room from weaning through adulthood (~ 50 days old). One week prior to intravenous catheter surgery, rats were handled daily and singly housed for the remainder of the experiment. Given the established sex differences in the acquisition and maintenance of cocaine self-administration and in the effects of intermittent access on behavior (Quigley et al. 2021; Algallal et al. 2020; Kawa and Robinson 2019) only one sex (males) was used in the current study. Future studies designed to assess potential differences in females should be conducted. All procedures were approved by the University of Michigan Committee on the Use and Care of Animals in accordance with AAALAC and AVMA guidelines.

### Intravenous catheter surgery

Rats underwent intravenous catheter surgery as described previously (Crombag et al. 2000; Ferrario et al. 2011). Briefly, rats were anesthetized using isoflurane (2–5% i.h. experiments 1 and 3) or a combination of ketamine (90 mg/kg, i.p.) and xylazine (10 mg/kg, i.p.; experiment 2). A silastic catheter was then inserted into the right jugular vein and passed beneath the skin over the right shoulder and placed in the mid-scapular region. Rats were given the analgesic carprofen immediately prior to surgery and for two days following surgery (5 mg/kg, s.c., one injection per day; Zoetis, NJ). Rats were allowed to recover for at least 5 days before training began. For the first 10 days following surgery, catheters were flushed daily with 0.2 ml sterile saline (Baxter, IL) containing 5 mg/ml gentamicin sulfate (Henry Schein, NY). For the remainder of the study, catheters were flushed with 0.2 ml sterile saline (Baxter, IL; experiments 1 and 2) or 0.2 mL of saline containing heparin (20 IU/mL; Millipore, MO; experiment 3) to prevent occlusions in the catheter. At the end of the experiment, catheters were tested for patency via intravenous infusion of 0.1 mL of methohexital sodium (10 mg/mL; JHP Pharmaceuticals, NJ). Rats that did not become ataxic immediately following the infusion were considered to have non-patent catheters and were removed from analyses as appropriate.

### Cocaine self-administration

Cocaine HCL was provided by the NIDA drug supply program. All training and testing were conducted in standard operant chambers (22 × 18 × 13 cm) located within sound-attenuating cabinets, with a ventilating fan (Med Associates, St Albans, VT, USA). Each chamber contained 2 nose poke ports located on the left and right side of one wall. One port was designated active and the other inactive (left/right counter-balanced across chambers). A red house light was located at the top center of the wall opposite the nose pokes, and a cue light was located above the active nose port. The house light was illuminated when rats were placed in the operant chambers and was turned off at the start of each session. During acquisition and IntA self-administration sessions, a fixed ratio 1 (FR1) schedule was used in which each response in the active port resulted in the delivery of an intravenous infusion of cocaine. Each infusion was paired with the illumination of a cue light for 5 s. During acquisition, there was a time-out period during which additional active responses were not reinforced for the duration the cue light was illuminated and for 15 s after (20 s in total). During IntA and threshold testing, another infusion could not be triggered until the cue light turned off (5 s). During initial training and IntA sessions, cocaine (0.4 mg/kg/infusion) was delivered in 50 μL over 2.6 s. Responses in the inactive port were recorded but had no consequence. The number of responses in the active and inactive ports, and infusions was recorded throughout each session using the Med Associates software. For all studies, rats were given one session per day, 7 days per week.

### Within session threshold procedure

Motivation for cocaine and preferred levels of cocaine intake were assessed using a within-session threshold procedure (Kawa et al. 2016; Carr et al. 2020). Briefly, during each session a response in the active port resulted in the delivery of an intravenous infusion of cocaine on an FR1 schedule. The dose of cocaine available decreased by a ¼ log every 10 min for a total of 120 min as follows (experiment 1, mg/kg/infusion: 1.28 0.72, 0.4, 0.23, 0.13, 0.072, 0.04, 0.023, 0.013, 0.007, 0.004, 0.002; experiment 2, μg/infusion: 383.5, 215.6, 121.3, 68.2, 38.3, 21.6, 12.1, 6.8, 3.8, 2.2, and 1.2). This effectively increases the price (i.e., work) needed to maintain the preferred level of cocaine consumption. Cocaine was given at specific concentrations, rather than adjusting to body weight, in the second experiment in an attempt to improve control and the potential to detect group differences. During these sessions, each infusion was paired with the illumination of the cue light for 5 s. Rats were tested in this way for at least 5 sessions and no more than 10 sessions, depending on the quality of curve fitting that resulted from these sessions (see demand analysis below).

### Intermittent access

Procedures here were similar to those described previously (Zimmer et al. 2012; Kawa et al. 2016; Carr et al. 2020). Each IntA session is comprised of 5-min drug available (DA) periods interspersed with 25-min no drug available (NDA) periods. This results in a binge-like pattern of cocaine intake, where brain concentrations of cocaine spike to a peak during DA periods and fall to baseline during the NDA periods prior to the start of the next DA period (Zimmer et al. 2012; Kawa et al. 2016; Kawa et al. 2019a, b). DA periods were signaled by turning off the house light, whereas NDA periods were signaled by illumination of the house light. During IntA sessions, each infusion was paired with the illumination of a cue light (5 s), and rats could take another infusion after the cue light turned off. After the 5-min DA period ended, the house light turned on, signaling the onset of a 25-min NDA period, during which active and inactive nose pokes were recorded but had no consequences. After the NDA period, the house light was again extinguished, signaling the onset of the next DA period. In experiments 1 and 2, each session consisted of 8 DA periods and 8 NDA periods (4 h total). In experiment 3, each session consisted of 7 DA periods and 7 NDA periods followed by an 8^th^ DA period in which a single self-administered infusion was followed by the final NDA period. This was done to assess locomotor activity, see below for additional details.

### Cocaine-seeking test

Fourteen days after the last IntA self-administration session (WD14) were given a single “cocaine-seeking” test (60 min). Rats were placed into the operant chambers with the house light illuminated (as during the NDA period of IntA), and the start of the test was signaled by extinguishing the house light. During this test, no cocaine was given but responses in the active nose port resulted in presentation of the previously drug-paired cue light (5 s) alone. Responses in the inactive nose port were recorded but had no consequences.

### c-Fos induction and immunohistochemistry

c-Fos expression induced by re-exposure to the previously cocaine-paired cue was assessed 14 days after the last IntA self-administration session. After 14 days of abstinence, rats were placed into the operant chambers with the house light illuminated, and the start of the test was signaled by turning off the house light. The session consisted of 10 presentations of the drug-paired cue once every minute for 5 s. Responses on the previously active and inactive nose poke were recorded but had no consequences. An additional set of drug-naïve rats were given 10 sessions in which the cue was presented as described above. The 10th session was conducted side-by-side with testing of the IntA group and brains were collected from all rats 90 min after the start of the session. Note, separate cohorts of rats were used for the cocaine-seeking test described above, and c-Fos induction (see also experiments 1 and 2 below).

Procedures for processing brains for immunohistochemistry (IHC) were similar to those described previously (Derman et al. 2020). Ninety minutes after the onset of the c-Fos induction test described above, rats were given sodium pentobarbital (Henry Schein, NY, 3.9 mg/mL, i.p.) and transcardially perfused with phosphate-buffered saline (PBS) followed by 4% paraformaldehyde (PFA, Sigma-Aldrich, MO) in PBS. Brains were extracted, placed into a 50/50 mix of 4% PFA and 30% sucrose solution, then stored at 4 °C. Twenty-four to forty-eight hours later, brains were transferred to a 30% sucrose solution and stored at 4 °C for 2–3 days. Next, coronal sections containing prefrontal cortex, striatum, and amygdala (30 μm) were made using a cryostat (Leica, Wetzlar, Germany) and stored at − 20 °C in cryoprotectant (50% 0.1 M phosphate buffer, 30% ethylene glycol, 30% sucrose) until they were processed for IHC.

All IHC was conducted at room temperature in free-floating sections placed on an orbital shaker (Talboys, NJ). Expression of c-Fos protein was determined using a Rabbit anti c-Fos primary antibody (CST, MA, #2250S) and an Alexa Fluor 555 Goat-anti-Rabbit secondary antibody (Invitrogen, MA, A32732), both at a 1:2000 dilution. All steps were conducted at room temperature. Briefly, sections were washed (12 × 10 min in 1X PBS), blocked (1.5 h, 1X PBS with 5% normal goat serum [MP Biomedicals, CA], 0.04% Triton-X [Sigma Aldrich, MO]), and incubated with primary antibody in blocking solution overnight (15–20 h). Slices were washed again (5 × 5 min in 1X PBS) and then incubated with secondary antibody in blocking solution (1.5 h). After a final set of washes (5 × 5 min in 1X PBS), tissue was mounted on Superfrost Plus microscope slides (Fisherbrand, MA) and cover-slipped with Prolong Gold and the nuclear fluorescent stain DAPI in the mounting medium (Invitrogen, MA, P36931) so that c-Fos counts could be normalized to total cell counts. An upright epifluorescence manual system microscope (Olympus, Tokyo, Japan, BX43) was used to image sections with an XM10 camera. Images were taken at 2 × and 10 × using cellSens software. Finally, c-Fos expression was quantified using standard background subtraction and thresholding procedures in ImageJ. The total number of cells was determined by counting the number of DAPI stained cells. The number of c-Fos positive cells were determined within the prelimbic and infralimbic regions of the medial prefrontal cortex (mPFC; AP: + 3.00 mm from bregma) and the NAc core and shell (AP: + 1.92 mm from bregma). Within the amygdala, c-Fos was measured in the anterior and posterior portions of the basolateral nucleus and the lateral amygdaloid nucleus (AP: − 2.5 mm from bregma). The number of c-Fos positive cells were normalized to the total number of cells within the sample region. Quantification was performed blind to group (OP vs OR) and treatment (naïve vs drug-experienced).

### Locomotor activity

Locomotor activity was examined using automated beam breaks (Med Associates, VT) within the self-administration chambers. Locomotor activity in response to a single self-administered infusion of cocaine (1.2 mg/kg, i.v.) was assessed after completion of the last NDA period of each IntA session (house light off during the infusion, as in DA periods), and in separate dedicated locomotor test sessions that were not immediately preceded by IntA. These separate test sessions took place within the self-administration chambers the day before IntA began and the day after IntA concluded. During these dedicated locomotor tests, rats were placed into the operant chamber with the house light illuminated. After 30 min of habituation, the house light was turned off and animals were allowed to self-administer a single infusion of cocaine. Following the single infusion, the house light was again illuminated and locomotor activity was measured via beam breaks for 25 min. For both procedures, cocaine infusion was accompanied by the illumination of the cue light (5 s).

### Statistics and analyses

Behavioral experiments were designed for within-subject and between-subject comparisons. Data were processed and organized with Microsoft Excel (Version 16.16.16) and statistical analyses were performed using GraphPad Prism (Version 9.0.0). Data were then analyzed using Student’s *t*-tests, one-way ANOVAs, repeated measures ANOVAs, GLMs, and mixed-effects models followed by Holm-Sidak’s and Tukey’s multiple comparisons tests as appropriate. Depending on the model selected, the degrees of freedom may have been adjusted to a non-integer value.

Data from within session threshold testing were examined using established curve fitting approaches (Newman and Ferrario 2020) to extract three main metrics of cocaine demand: *Q*_0_, the preferred level of consumption when the price of cocaine is negligible; *P*_max_, the price at which maximum work is performed; and *R*_max_, the maximum work performed to obtain cocaine regardless of price. Fundamental to the assessment of cocaine demand (i.e., the consumption of cocaine as a function of the price to obtain it) is that cocaine consumption must be relatively flat when cost is low, and then decline to zero as cost increases (Bentzley et al. 2013; Hursh and Silberberg 2008). For demand analysis, data from each within threshold test session were plotted for individual rats and only sessions that followed the pattern described above were included in demand analysis (see also Fig. 3A for examples). If an animal had no sessions that followed the pattern, they were excluded from the demand analysis; this was the case for 1 out of 12 rats in the OP group and 2 out of 12 rats in the OR group at the Post IntA time point only; experiment 1. Data from sessions were fit using the model described by Newman and Ferrario (2020) and fits were then confirmed visually. *P*_max_, *R*_max_, and *Q*_0_ values obtained from these fits were then averaged across several sessions for each rat.

#### Experiment 1

The first set of studies set out to evaluate potential inherent differences in cocaine demand between OP and OR groups, the effect of IntA cocaine self-administration on cocaine demand in these groups, and potential differences in cocaine-seeking following withdrawal. For these studies, OP (*n* = 12) and OR (*n* = 14) groups were trained to self-administer cocaine as described above (6 sessions; 1 h or a maximum of 20 infusions/session). Next, baseline cocaine demand was assessed using within session threshold procedure described above (5–10 sessions). This was followed by IntA self-administration (12 sessions) and post cocaine demand assessment (5–10 sessions). All rats then underwent a 14-day period of forced abstinence in which they remained in their home cages followed by a single cocaine-seeking test.

#### Experiment 2

The goal of experiment 2 was to further evaluate demand for cocaine before and after IntA, and to examine potential differences in cue-induced c-Fos induction in obesity-prone (*n* = 8) vs obesity-resistant (*n* = 8) rats. To avoid potential differences in drug exposure and number of CS-US pairings during acquisition, an infusion criterion (IC) procedure was used (Saunders and Robinson 2010; Kawa et al. 2016). Briefly, these sessions terminated once the rat obtained a set number of infusions, or after one hour elapsed. All rats received 2 sessions at IC10, 3 at IC20, and 3 at IC40. Next, baseline cocaine demand was assessed using a within session threshold procedure (5–10 sessions), followed by IntA self-administration (8 sessions), and post IntA threshold testing (2 sessions). Finally, rats underwent a 14-day period of forced abstinence followed by cue-induced c-Fos induction. Additional drug naïve controls were included (OP *n* = 5 OR *n* = 5), as described above.

#### Experiment 3

The goal of this final experiment was to evaluate cocaine-induced locomotor sensitization in obesity-prone (*n* = 11) vs obesity-resistant (*n* = 12) rats across IntA. Rats here were trained to self-administer cocaine as described in experiment 1. Following acquisition, the acute locomotor response to a single self-administered infusion of cocaine was assessed (Baseline). Next, locomotor activity to a single self-administered infusion of cocaine was assessed at the end of each IntA session (12 sessions total). Finally, locomotor activity was assessed 24 h after the last IntA session (post).

## Results

### Exp 1: Does demand for cocaine differ between obesity-prone and obesity-resistant groups?

#### Self-administration acquisition

Figure 1A shows the timeline for experiment 1 followed by the average number of infusions (Fig. 1B) and active vs inactive responses (Fig. 1C) during initial acquisition. The number of infusions increased similarly in OP and OR groups across the 6 training sessions (Fig. 1B: two-way RM ANOVA; main effect of session: *F*(2.96, 73.99) = 15.06, *p* < 0.01; no main effect of strain: *F*(1, 25) = 2.43, *p* = 0.13; no strain × session interaction; *F*(5, 125) = 1.83, *p* = 0.11). In addition, both groups learned to discriminate between the active and inactive nose poke ports, with responding on the active port increasing across sessions, and inactive responses remaining low and stable (Fig. 1C: three-way RM ANOVA; main effect of port; *F*(1, 26) = 40.11, *p* < 0.01; main effect of session; *F*(2.04, 53.11) = 4.86, *p* = 0.01; significant session × port interaction; *F*(5, 115) = 3.74, *p* < 0.01). No differences in responding or number of infusions were observed between groups.

#### IntA self-administration

**Fig. 2:**
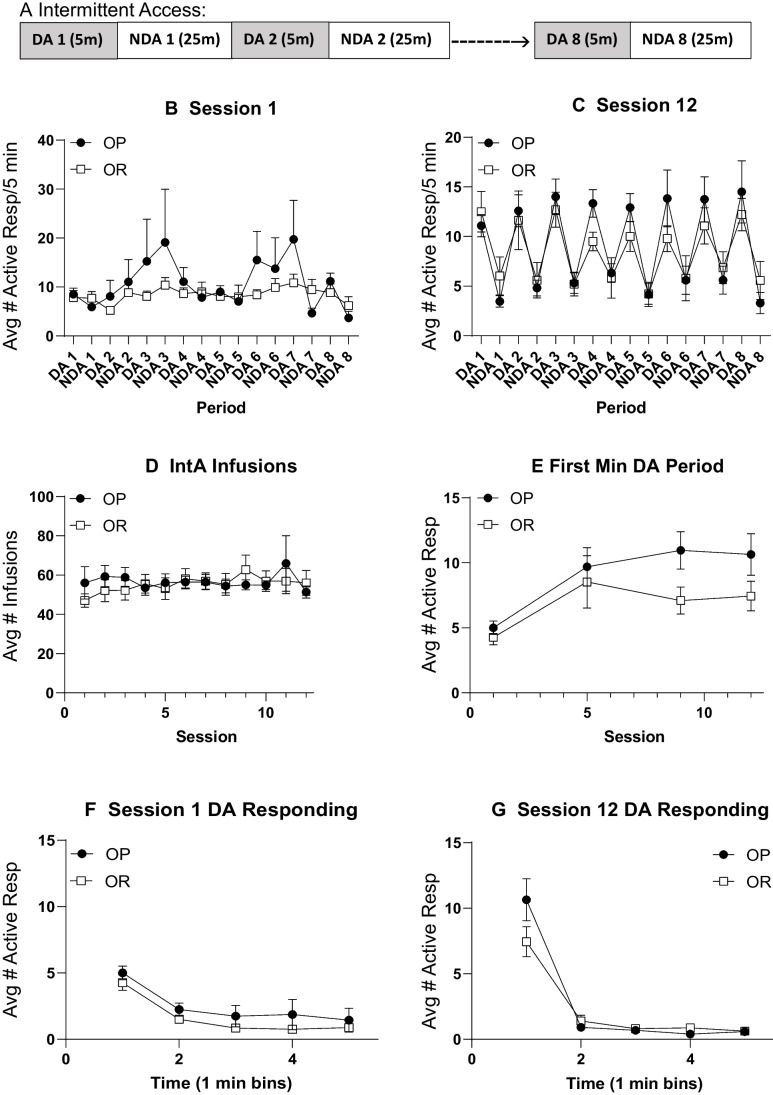
IntA cocaine self-administration is similar between groups and results in escalation of drug intake. **A** Schematic of alternating drug available (DA) and no drug available (NDA) periods during each IntA session. **B** Active responses across DA and NDA periods during the first IntA session in obesity-prone and obesity-resistant groups. Neither group showed discrimination between the DA and NDA periods. **C** Active responses across DA and NDA periods during the last IntA session. By the 12th session, both groups showed strong discrimination, making more active responses during the DA than the NDA period. **D** Average infusions taken across each IntA session was similar between groups. **E** Active responses during the first minute of the DA period increased across IntA in both groups. **F** Active responses during each min of the 5-min DA period during session 1. **G** Active responses during each min of the 5-min DA period during session 12. While both groups escalated their intake from the first to last session, no group differences were found

Figure 2A outlines the pattern of drug available (DA) and no drug available (NDA) periods during an IntA session. During IntA, responses in the inactive port were low and stable throughout all sessions and were similar between OP and OR groups (Supplemental Fig. 1A: three-way RM ANOVA; no main effect of strain; *F*(1, 24) = 1.81, *p* = 0.19; no strain × session interaction; *F*(11, 264) = 0.98, *p* = 0.46; no strain × port interaction; *F*(1, 24) = 0.10, *p* = 0.76; Avg inactive response over 12 sessions = 2–23 responses ± 1.50 SEM). Active responses across each DA and NDA period for the first and last IntA session are shown in Fig. 2B and C, respectively. During the first IntA session, neither group showed strong discrimination between alternating DA and NDA periods (Fig. 2B: two-way RM ANOVA; no main effect of period; *F*(1.53, 36.6) = 2.19, *p* = 0.14). However, by session 12, both groups showed strong discrimination, responding more during the DA period than the NDA period (Fig. 2C: two-way RM ANOVA; main effect of period; *F*(4.31, 103.5) = 20.87*, p* < 0.01). In addition, active responding was similar in OP and OR groups, and there were no group differences in the number of infusions taken across IntA sessions (Fig. 2D: two-way RM ANOVA; no main effect of strain; *F*(1, 24) = 0.04, *p* = 0.84).

Figure 2E shows the average number of active responses during the first minute of the DA period for each session. Active responses escalated across IntA sessions in both groups (Fig. 2E: two-way RM ANOVA; main effect of session; *F*(2.44, 58.63) = 10.53, *p* < 0.01), and no group differences were found (Fig. 2E: two-way RM ANOVA; no main effect of strain; *F*(1, 24) = 2.38, *p* = 0.14; no significant session × strain interaction; *F*(3, 72) = 1.26, *p* = 0.30). In addition, active responding was greatest during the first minute (M1) of the DA period compared to the remaining 4 min during both session 1 and session 12 for both groups (Fig. 2E, F). Overall, IntA produced escalation and binge-like patterns of cocaine intake that was similar in OP and OR groups.

#### Demand for cocaine

**Fig. 3:**
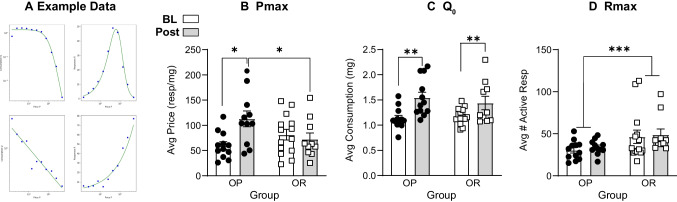
Cocaine demand testing using the within session threshold procedure. **A** Example cocaine intake (closed circles) and the corresponding fitted curve (green line) from one demand session in two separate rats. In the upper panel, cocaine consumption was relatively flat at low cost and declined to zero as cost increased and thus curve fitting can provide a reliable measure of *Q*_0_, *P*_max_, and *R*_max_. The lower panel shows intake from a demand session that did not follow this pattern, and thus cannot be used to determine these metrics. **B** Motivation to take cocaine (*P*_max_) at baseline (BL; OP *n* = 12; OR *n* = 14) and after 12 sessions of IntA cocaine self-administration (post; OP *n* = 11; OR *n* = 10). *P*_max_ was increased following IntA in the obesity-prone, but not obesity-resistant group. **C** The preferred level of cocaine intake (*Q*_0_) did not differ between groups but increased in both groups following IntA. **D** *R*_max_ was greater in the obesity-resistant vs obesity-prone group at both timepoints and was not affected by IntA. * = Sidak’s post-test *p* < 0.05; ** = significant main effect of test; *** = significant main effect of strain

Figure 3A shows cocaine intake (closed circles) and the corresponding fitted curve (blue line) from one demand session in two separate rats. In the upper panel, cocaine consumption was relatively flat at low cost and declined to zero as cost increased. Thus, curve fitting can provide a reliable measure of *Q*_0_ and *P*_max_. The lower panel shows data from a demand session that did not follow this pattern, and thus cannot be used to determine these metrics. The majority of rats tested showed a typical demand curve for 3 or 4 out of 5 demand sessions. This is expected, as there is some learning that takes place when the session parameters change (e.g., no NDA period, changes in dose available). There were a small minority of rats (1/12 OP and 2/12 OR rats) that never showed the expected demand pattern despite additional testing (up to 10 sessions). Data from these animals was not included in analyses (see also methods).

Figure 3B shows *P*_max_ before (baseline; BL) and after IntA (post). Motivation to take cocaine (*P*_max_) did not differ between groups at baseline, but was significantly increased following IntA in OP, but not OR groups (Fig. 3B: two-way RM ANOVA; significant test × strain interaction; *F*(1, 18) = 9.19, *p* < 0.01, Sidak’s post-test; OP vs OR baseline, *p* = 0.42; OP baseline vs post IntA, *p* < 0.01, OR baseline vs post IntA, *p* = 0.80). Consistent with this, *P*_max_ was also greater in OP vs OR groups after IntA (Fig. 3b: Sidak’s post-test; OP vs OR post, *p* = 0.04).

Figure 3C shows the preferred level of consumption (*Q*_0_). No group differences in *Q*_0_ were found, but IntA significantly increased *Q*_0_ in both groups (Fig. 3C: two-way RM ANOVA; no main effect of strain; *F*(1, 25) = 0.12, *p* = 0.73; significant main effect of test; *F*(1, 19) = 24.81, *p* < 0.01, no significant test × strain interaction; *F*(1, 19) = 0.78, *p* = 0.39).

Figure 3D shows the maximum work performed to obtain cocaine regardless of price (*R*_max_). *R*_max_ was significantly greater in OR vs OP groups at both timepoints (Fig. 3D: two-way RM ANOVA; main effect of strain; *F*(1, 25) = 4.57, *p* = 0.04), but did not change as a function of IntA in either group (Fig. 3D: two-way RM ANOVA; no main effect of test; *F*(1, 18) = 3.49, *p* = 0.08, no significant test × strain interaction; *F*(1,18) = 0.40, *p* = 0.54). In sum, demand testing revealed enhancements in motivation for cocaine in OP, but not OR groups following IntA; this was not related to the preferred level of cocaine consumption or maximal active nose poke responding.

#### Cocaine-seeking test

**Fig. 4:**
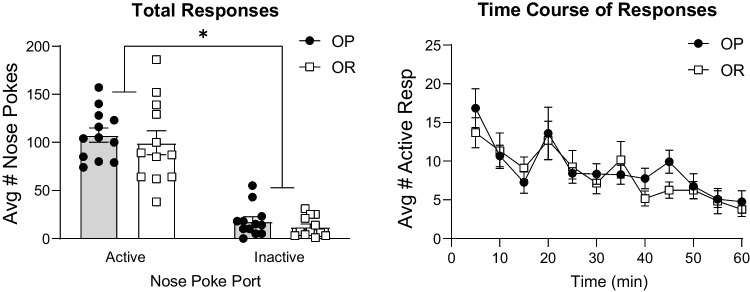
Cocaine-seeking when responding was reinforced by the cue alone was similar between groups. **A** Total active and inactive responses in obesity-prone (*n* = 12) and obesity-resistant (*n* = 12) groups. **B** Time course of active responding in both groups. * = significant main effect of nose poke port

Figure 4 shows active and inactive nose poke responding during the WD14 cocaine-seeking testing. No cocaine was given, but responses in the active nose port resulted in presentation of the previously cocaine paired cue. Figure 4B shows the time course of active responding during this test. Both OP and OR groups maintained active vs inactive discrimination, responding more on the active nose poke than the inactive but no group differences were found (Fig. 4A; two-way RM ANOVA; main effect of nose poke port; *F*(1, 22) = 149.7, *p* < 0.01; no main effect of strain; *F*(1, 22) = 0.67, *p* = 0.42, no significant port × strain interaction; *F*(1, 22) = 0.02, *p* = 0.88).

### Exp 2: Does cue-induced c-Fos expression differ between obesity-prone and obesity-resistant groups?

#### Self-administration acquisition

**Fig. 5:**
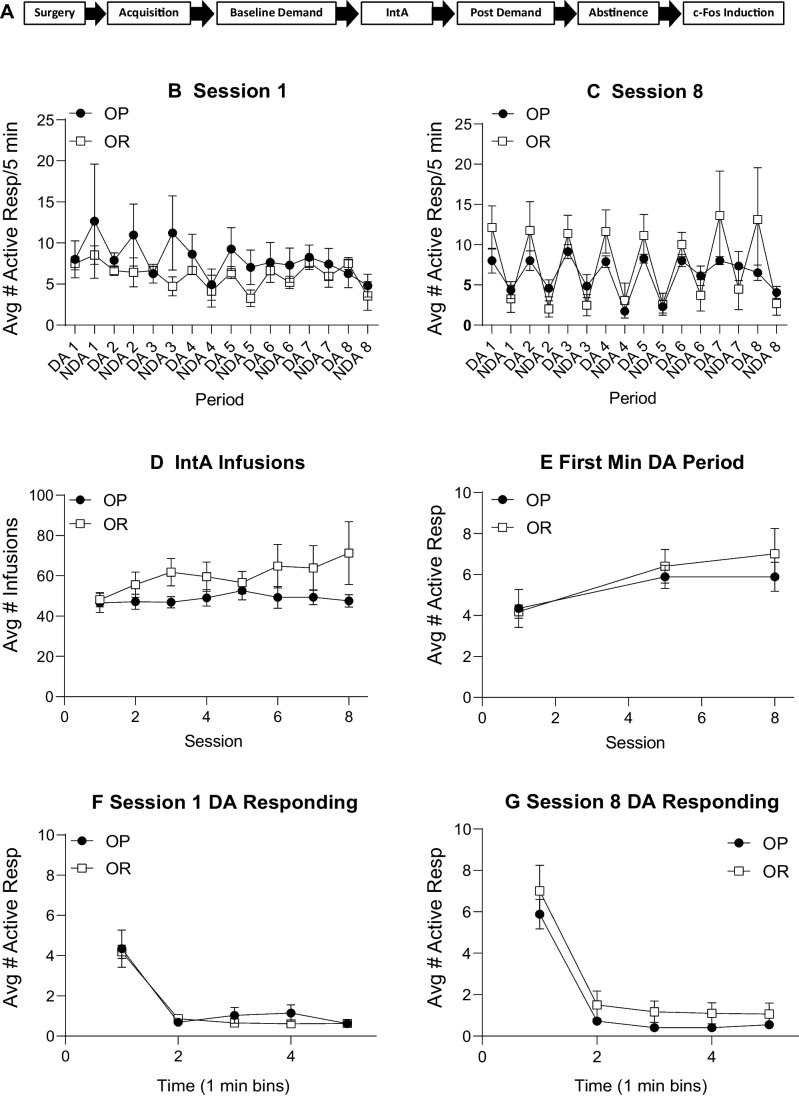
Experiment 2; IntA cocaine self-administration is again similar between groups. **A** Timeline for experiment 2. **B** Active responses across DA and NDA periods during the first IntA session in obesity-prone (*n* = 8) and obesity-resistant (*n* = 8) groups. Neither group showed discrimination between the DA and NDA periods. **C** Active responses across DA and NDA periods during the last IntA session. By the 8th session, both groups showed strong discrimination, making more active responses during the DA than the NDA period. **D** Infusions taken across each IntA session was similar between groups. **E** Active responses during the first minute of the DA period increased across IntA in both groups. **F** Active responses during each min of the 5-min DA period during session 1. **G** Active responses during each min of the 5-min DA period during session 12. While both groups escalated their intake from the first to last session, no group differences were found

Figure 5A shows the timeline for experiment 2. To control for initial drug and cue experience during acquisition, an infusion criterion procedure was used. Thus, there were no group differences in initial cocaine intake (data not shown). In addition, both groups learned to discriminate between the active and inactive nose poke ports with responding on the active port increasing across sessions and inactive responding remaining low (data not shown; three-way RM ANOVA; main effect of nose poke port; *F*(1, 14) = 43.8, *p* < 0.01; significant session × nose poke port interaction; *F*(7,96) = 2.13, *p* = 0.04) and no differences between groups (three-way RM ANOVA; no main effect of strain; *F*(1, 14) = 0.76, *p* = 0.40).

#### IntA self-administration

During IntA, responses in the inactive port remained low and stable throughout all 8 sessions and there were no differences between OP and OR groups (Supplemental Fig. 1B; three-way RM ANOVA; no main effect of strain; *F*(1, 97) = 0.63, *p* = 0.43; no strain × session interaction; *F*(17, 97) = 1.46, *p* = 0.19; no strain × port interaction; *F*(1, 97) = 0.15, *p* = 0.23; Avg inactive response across 12 sessions = 0.25–11.13 responses ± 1 SEM). As in Exp. 1, neither group discriminated between the DA and NDA periods during the first IntA session (Fig. 5B: two-way RM ANOVA; no main effect of period; *F*(2.86, 39.99) = 1.52, *p* = 0.23), but showed discrimination between the two periods during the last session, responding more during the DA versus the NDA period (Fig. 5C: two-way RM ANOVA; main effect of period; *F*(2.12, 29.61) = 12.07*, p* < 0.01). There were no group differences in the number of infusions taken across IntA sessions (Fig. 5D: two-way RM ANOVA; no main effect of strain; *F*(1, 14) = 1.90, *p* = 0.19; no session × strain interaction; *F*(7, 97) = 1.59, *p* = 0.15).

Figure 5E shows the average number of active responses during the first minute of the DA period for each session. Active responses escalated across IntA sessions in both groups, and no group differences were found (Fig. 5E: two-way RM ANOVA; main effect of session; *F*(1.60, 22.38) = 6.56, *p* < 0.01; no main effect of strain; *F*(1, 14) = 0.32, *p* = 0.58; no group × session interaction; *F*(2, 23) = 0.48, *p* = 0.62). In addition, active responding was the greatest during the first minute (M1) of the DA period compared to the remaining 4 min during both the first and last IntA session for both groups (Fig. 5F, G). Overall, the pattern of drug intake and active responding was similar to that seen in experiment 1, with rats in both groups escalating their drug intake and consuming the majority of cocaine within the first minute of each DA period.

#### Demand for cocaine

Rats in Exp. 2 also underwent demand testing before and after IntA experience. For this set of studies cocaine concentration was not adjusted for body weight, and instead was given at decreasing concentration to vary price (see methods). In addition, rats only received two sessions of demand testing after IntA. Unfortunately, this approach resulted in behavior that did not show the expected consumption pattern (flat consumption at low price that declines to zero as price increases). Thus, these data could not be used to evaluate motivation as planned and are therefore not included here. It is important to note though that cocaine intake was similar across groups during these sessions.

#### Cue-induced c-Fos expression

**Fig. 6:**
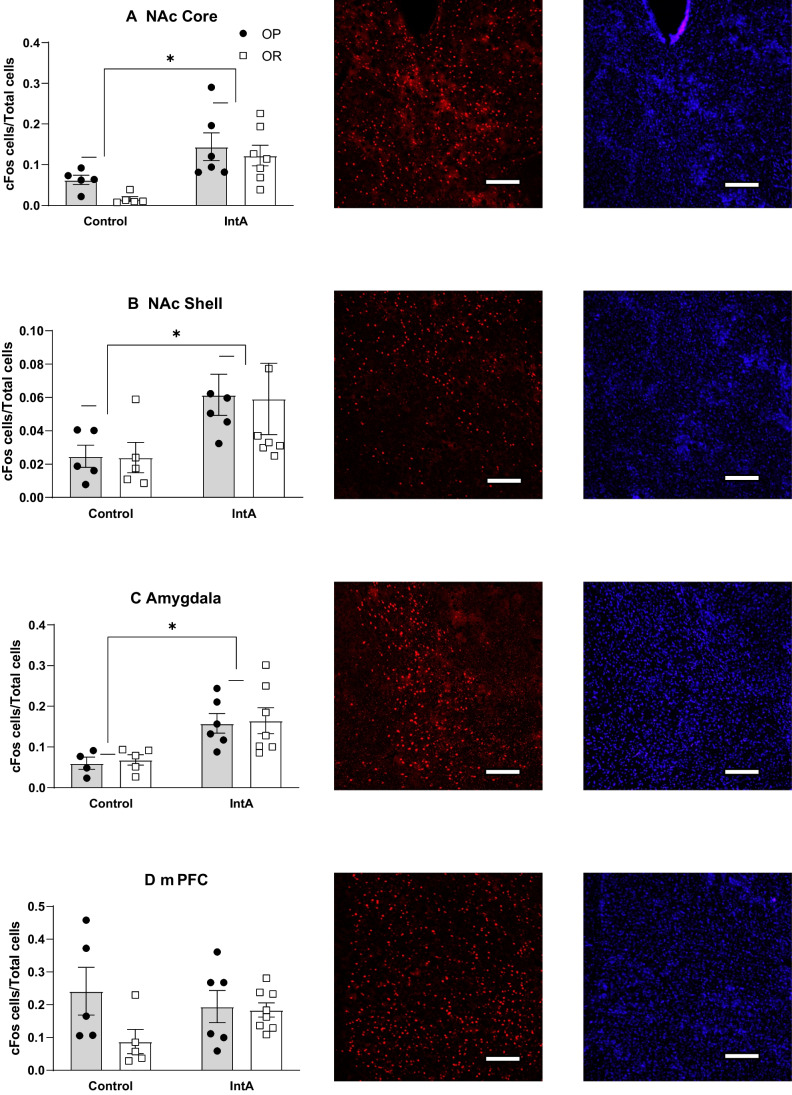
Cue-induced c-Fos expression in the NAc core (**A**), NAc shell (**B**), amgydala (**C**), and mPFC (**D**). Representative c-Fos (red) and DAPI (blue) images are shown to the right of each bar graph; scale bar = 100 microns. No differences between obesity-prone and obesity-resistant groups were found for any measure. However, cue presentation resulted in greater c-Fos expression in IntA vs control groups in the NAc core, NAc shell and amygdala. * = significant main effect of treatment

Fourteen days after the last cocaine self-administration session, cue-induced c-Fos expression was assessed in the NAc, amygdala and mPFC following non-contingent exposure to the previously cocaine-paired cue. Controls were drug-naive and exposed to non-contingent cue presentations (see methods). Rats were free to respond in the nosepoke ports during this session, but responses in the previously active port had no consequences (see also Supplemental Fig. 2). Effects on c-Fos expression were similar in sub-regions of the mPFC and amygdala and were therefore collapsed to simplify data presentation. Figure 6 shows c-Fos expression normalized to total cell number in the NAc core (A), NAc shell (B), amygdala (C) and mPFC (D). No differences between OP and OR groups were found for any measure. However, cue presentation resulted in greater c-Fos expression in IntA vs control groups in the NAc core (Fig. 6A: two-way RM ANOVA; main effect of treatment; *F*(1, 19) = 14.61, *p* < 0.01), NAc shell (Fig. 6B: two-way RM ANOVA; main effect of treatment; *F*(1, 19) = 5.30, *p* = 0.03), and the amygdala (Fig. 6C: two-way RM ANOVA; main effect of treatment; *F*(1, 18) = 13.64, *p* < 0.01). In contrast, there was no difference in mPFC c-Fos expression between control and IntA groups (Fig. 6C: two-way RM ANOVA; no main effect of treatment; *F*(1, 20) = 0.30, *p* = 0.59). Thus, the previously drug-paired cue produced robust activation in the NAc and amygdala but not mPFC that was comparable between groups.

### Exp 3: Does locomotor sensitization to self-administered cocaine differ between obesity-prone and obesity-resistant groups?

#### Self-administration acquisition

**Fig. 7:**
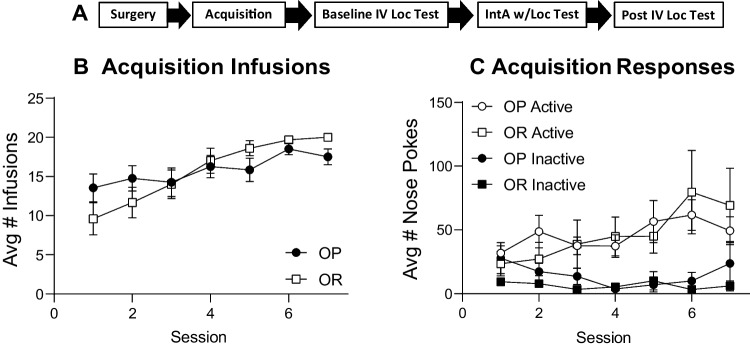
Experiment 3; acquisition of cocaine self-administration was similar between groups. **A** Timeline for experiment 3. **B** The number of infusions across each session in obesity-prone (OP, *n* = 12) and obesity-resistant (OR, *n* = 12) groups. **C** Average active and inactive responding across each session

Figure 7A shows the timeline for experiment 3 followed by the average number of infusions (Fig. 7B) and active vs inactive responses (Fig. 7C) during initial acquisition. The number of infusions increased similarly in OP and OR groups across the 7 training sessions (Fig. 7B: two-way RM ANOVA; main effect of session; *F*(3.37, 73.66) = 15.59, *p* < 0.01; no main effect of strain; *F*(1, 22) = 0.01, *p* = 0.97). In addition, both groups learned to discriminate between the active nose and inactive nose poke ports (Fig. 7C: three-way RM ANOVA; main effect of nose poke port; *F*(1, 22) = 11.5*, p* < 0.01), with no group differences in active or inactive responding (Fig. 7C: two-way RM ANOVA; active: no main effect of strain; *F*(1, 22) = 0.01, *p* = 0.97; inactive: no main effect of strain; *F*(1, 22) = 1.13, *p* = 0.30).

#### IntA self-administration

**Fig. 8:**
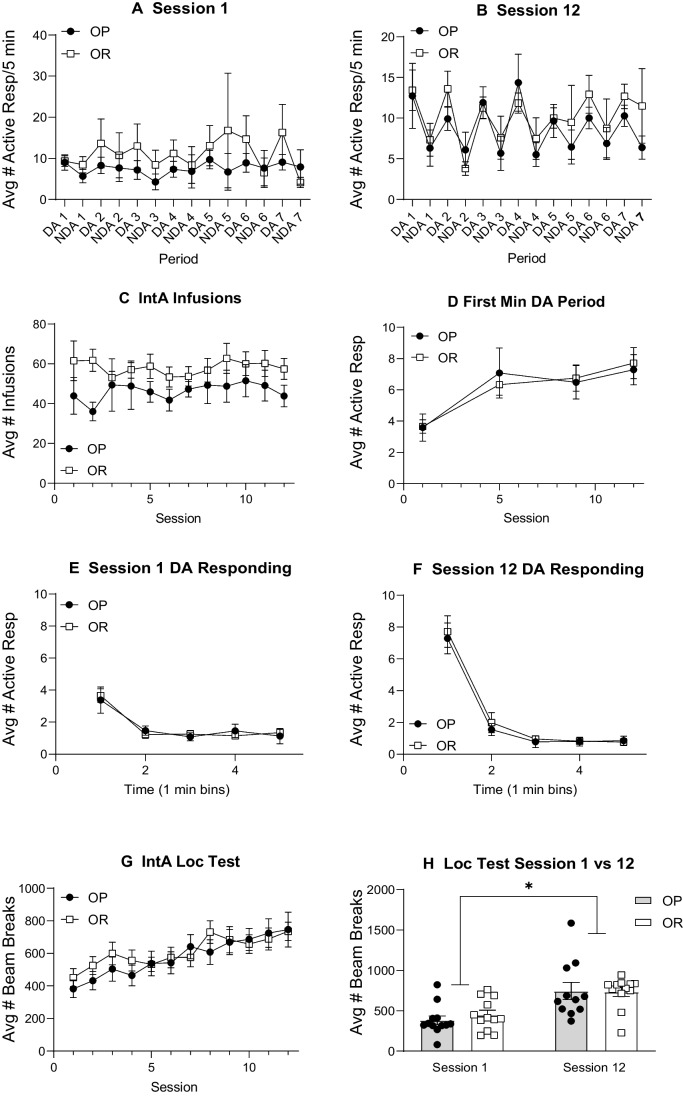
IntA cocaine self-administration results in locomotor sensitization that is similar between groups. **A** Active responses across DA and NDA periods during the first IntA session in obesity-prone (*n* = 11) and obesity-resistant (*n* = 12) groups. **B** Active responses across DA and NDA periods during the last IntA session. Again, both groups showed strong discrimination, making more active responses during the DA than the NDA period. **C** Infusions taken across each IntA session was similar between groups. **D** Active responses during the first minute of the DA period increased across IntA in both groups. **E** Active responses during each min of the 5-min DA period during session 1. **F** Active responses during each min of the 5-min DA period during session 12. While both groups escalated their intake from the first to last session, no group differences were found. **G** Locomotor activity in response to a single self-administered infusion of cocaine taken at the end of each IntA session. **H** Total locomotor activity in response to a single self-administered infusion of cocaine at the end of the first and last Int A session. IntA experience produces progressive increase in locomotor activity in response to the same dose of cocaine (1.2 mg/kg, i.v.). * = significant main effect of session

During IntA, responses in the inactive port were low and stable throughout all 12 sessions and there were no differences in responding between OP and OR groups (Supplemental Fig. 1C: three-way RM ANOVA; no main effect of strain; *F*(1, 21) = 0.82, *p* = 0.38; no strain × session interaction; *F*(11, 231) = 0.99, *p* = 0.45; no strain × nose poke port interaction; *F*(1, 21) = 0.21, *p* = 0.65; Average inactive response across 12 sessions = 2–28 responses ± 1.55 SEM). Neither group discriminated between the DA and the NDA period during the first IntA session (Fig. 8A: two-way RM ANOVA; no main effect of period; *F*(1.81, 36.27) = 1.19, *p* = 0.31). However, by session 12, both groups showed clear discrimination, responding more on the active nose poke during the DA than the NDA period (Fig. 8B: two-way RM ANOVA; main effect of period; *F*(3.12, 65.33) = 3.96*, p* = 0.01). In addition, there were no group differences in the number of infusions taken during IntA (Fig. 8C: two-way RM ANOVA; no main effect of strain; *F*(1, 22) = 2.17, *p* = 0.16). Figure 8D shows the average number of active responses during the first minute of the DA period for each session. As above, both groups escalated their drug intake during the first minute of the DA period across the 12 sessions (Fig. 8D: two-way RM ANOVA; main effect of session; *F*(2.52, 50.29) = 8.92, *p* < 0.01; no main effect of strain; *F*(1, 21) = 0.01, *p* = 0.97). Furthermore, active responding was greatest during the first minute (M1) of the DA period compared to the remaining 4 min during both session 1 and session 12 for both groups (Fig. 8E, F).

#### Locomotor sensitization

Figure 8G shows locomotor activity in response to self-administered cocaine taken in the last DA period of each IntA session. Locomotor activity increased significantly across IntA sessions with a similar magnitude of increase in both OP and OR groups (Fig. 8G: two-way RM ANOVA; main effect of session; *F*(4.90, 106.4) = 9.19, *p* < 0.01; no main effect of strain; *F*(1, 22) = 0.26, *p* = 0.61). Summary data comparing locomotor activity during session 1 and 12 are shown in Fig. 8H (two-way RM ANOVA; main effect of session; *F*(1, 21) = 24.69, *p* < 0.01; no main effect of strain; *F*(1, 22) = 0.16, *p* = 0.70; no strain × session interaction; *F*(11, 239) = 0.78, *p* = 0.67).

**Fig. 9:**
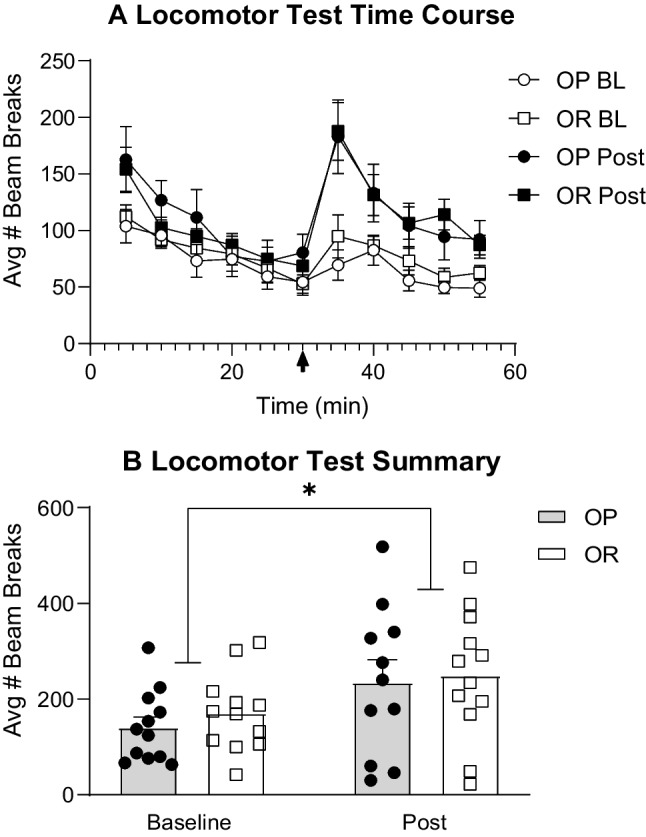
Cocaine-induced locomotor activity before and after IntA experience is similar between groups and sensitizes across time. **A** Time course of locomotor activity across habituation and after a single self-administered infusion of cocaine (1.2 mg/kg, i.v.) after acquisition but prior to IntA (baseline, BL) and 24 h after the last IntA session (post). **B** Summary of cocaine-induced locomotor activity. IntA experience produced a similar degree of locomotor sensitization in both groups. * = significant main effect of test

Figure 9 shows locomotor activity in response to self-administered cocaine assessed at baseline (after acquisition but prior to IntA) and the day after the last IntA session (post), with time course data shown in panel A and summary data shown in panel B. IntA resulted in a sensitized locomotor response to cocaine in both groups, with a greater number of beam breaks during post vs baseline testing (Fig. 9B: two-way RM ANOVA; main effect of test; *F*(1, 21) = 12.85, *p* < 0.01; no main effect of group; *F*(1, 22) = 0.31, *p* = 0.58).

## Discussion

Obesity-prone rats show greater responsivity to food cues and greater sensitivity to neural plasticity induced by sugary, fatty food than do obesity-resistant rats (see Ferrario 2020 for review). Given the overlap between neural circuits that regulate motivation for food and addictive drugs like cocaine, the current study examined whether obesity-prone rats may also be more sensitive to cocaine-induced neurobehavioral plasticity and more responsive to cocaine-paired cues than obesity-resistant rats. We found that obesity-prone and obesity-resistant groups did not differ in their initial acquisition of cocaine self-administration, baseline demand for cocaine or their locomotor response to self-administered cocaine. Furthermore, after IntA self-administration, responding in the absence of cocaine but the presence of a previously cocaine-paired cue was similar in both groups. In addition, there was robust cue-induced c-Fos expression in the NAc and amygdala after IntA, and locomotor sensitization across IntA sessions. However, the magnitude of these effects was similar across obesity-prone and obesity-resistant groups. Overall data here are in striking contrast to established inherent behavioral differences in motivation for food and responsivity to food cues between obesity-prone and obesity-resistant lines (discussed further below).

### Acquisition and baseline demand for cocaine

We did not find any differences in acquisition of cocaine self-administration between obesity-prone and obesity-resistant rats (Figs. 1, 7). Specifically, in all studies, discrimination between the active and inactive nose poke ports emerged similarly across the groups during initial training, and drug intake was comparable between groups. In addition, during baseline demand testing the preferred level of cocaine consumption at low price (*Q*_0_) was similar between groups. The absence of group differences in learning to self-administer cocaine is consistent with prior studies of instrumental responding for food in these lines. However, when the effort needed to obtain food is low (FR1, FR3), obesity-prone rats show enhanced responding compared to obesity-resistant rats that results in greater food intake (Vollbrecht et al. 2015). Thus, although consumption of food and effort to obtain it are greater in obesity-prone vs obesity-resistant rats, no such pattern was found for intravenous cocaine. It is possible that differences in acquisition of cocaine self-administration were masked by the dose of drug used here. However, baseline demand data suggest that this is not the case, as *Q*_0_ was similar between groups (Fig. 3C). The only group difference found at baseline was in *R*_max_, which was greater in obesity-resistant vs obesity-prone rats (Fig. 3D). This difference was maintained after IntA. Although *R*_max_ was greater in obesity-resistant groups, measures that rely on rates of responding must be interpreted cautiously given the stimulant properties of cocaine.

### Effects of intermittent access

We used an IntA procedure here because this induces robust neural and behavioral plasticity associated with drug addiction (Kawa et al. 2016; Samaha et al. 2021). No differences in behavior during IntA were found between groups across all three studies (Figs. 2, 5, and 8). However, as expected, rats did show good discrimination between drug available, and no drug available periods. In addition, effects of IntA on behavior were overall similar to previous reports, with rats consuming the majority of their infusions during the first minute of the drug available period, and this intake escalating across sessions (Kawa et al. 2016; Allain et al. 2018; Kawa and Robinson 2019).

The main point of divergence between the groups was in the effect of IntA on motivation for cocaine, where IntA experience increased *P*_max_ in obesity-prone, but not obesity-resistant groups (Fig. 3B). This effect of IntA in obesity-prone rats is consistent with prior studies showing that IntA increases *P*_max_ and other measures of motivation in outbred Sprague Dawley rats (Calipari et al. 2013; Kawa et al. 2019a, b; James et al. 2019), the same strain from which rats in the current study were originally derived. Thus, it is the absence of effects in obesity-resistant rats that deviates from established effects of IntA on cocaine motivation. It is possible that obesity-resistant rats are less sensitive to cocaine-induced plasticity that underlies these enhancements in motivation, and/or that neuroplasticity occurs in obesity-resistant rats that opposes the effects of cocaine on motivation.

Importantly, differences in motivation for drug between obesity-prone and obesity-resistant groups was not caused by differences in IntA experience, as there were no group differences in the number of infusions taken or escalation of intake over the 12 IntA sessions (Fig. 2). In addition, while *P*_max_ increased only in the obesity-prone group, *Q*_0_ increased similarly in both groups following IntA. To our knowledge, this is the first time that an increase in *Q*_0_ has been observed following IntA; this provides additional evidence for dissociations between hedonic vs motivational properties of cocaine.

In regard to potential differences in response to drug-paired cues, there were no differences in the ability of a previously drug-paired cue to maintain drug-seeking behavior in the absence of cocaine between groups (Fig. 4). Furthermore, while passive exposure to the cocaine-cue induced robust c-Fos expression in the NAc and amygdala compared to controls, the magnitude of these effects was again similar across groups (Fig. 6). Thus, there is no evidence for enhanced sensitivity to cocaine-paired cues in obesity-prone vs –resistant rats after IntA experience. The conditions used here are typical of many studies in terms of number of sessions, cocaine intake and cue exposures. However, it is possible that differences could emerge after longer periods of withdrawal or in response to different self-administration conditions (e.g., more sessions, or long access sessions). Nonetheless, these results are in stark contrast to enhanced responsivity to food cues in obesity-susceptible compared to obesity-resistant rats (Robinson et al. 2015; Derman and Ferrario 2018; Alonso-Caraballo and Ferrario 2019). Finally, the pattern of cocaine cue-induced c-Fos expression found here is consistent with previous reports (Brown et al. 1992; Neisewander et al. 2000; Hotsenpiller et al. 2002; Kufahl et al. 2009). One caveat to this is the absence of cue-induced c-Fos in the mPFC here (Fig. 6D). Interestingly, this was due to relatively high c-Fos expression in drug-naïve obesity-prone controls.

Another feature associated with IntA and plasticity of mesolimbic systems is the development of robust psychomotor sensitization (Carr et al. 2020; Allain et al. 2017, 2021; Wolf and Ferrario 2010). Here, we measured locomotor activity induced by self-administration of a single bolus of cocaine (1.2 mg/kg) at the end of each IntA session (Fig. 8) and prior to vs after IntA (Fig. 9). In both cases, locomotor sensitization developed across the 12 sessions of IntA, and the magnitude of this increase was similar in obesity-prone and obesity-resistant groups. Furthermore, cocaine-induced locomotor activity before IntA experience was also similar between groups (Fig. 9a). While consistent with the data discussed above, these results differ from previous studies showing enhanced acute locomotor activity in response to cocaine, and stronger expression of cocaine-induced locomotor sensitization in obesity-prone vs obesity-resistant rats (Oginsky et al. 2015; Vollbrecht et al. 2015, 2016). There are many potential explanations for this difference, most notably route of administration (i.v. here vs i.p. in prior reports) and the doses used. Additionally, the use of the IntA procedure, which produces robust sensitization, may have produced a ceiling effect thereby limiting our ability to detect group differences. However, our goal was not to identify conditions that do or do not produce behavioral differences between our groups. Rather, our aim was to use a procedure that induces neural and behavioral plasticity specifically related to the development of addiction and ask whether or not under those conditions we observe differences between obesity-prone and -resistant groups.

Overall, the pattern of neural and behavioral results here do not support the idea that there are robust differences in neurobehavioral responses to cocaine or cocaine-paired cues in obesity-prone vs obesity-resistant rats. Thus, behavioral differences that exist between obesity-prone and -resistant animals for food and food cues do not necessarily transfer to drugs and drug-paired cues. Furthermore, neural and behavioral plasticity induced by IntA were either similar in both groups, or commensurate with previous reports in “standard” outbred rats. These data highlight the need to carefully consider potential differences between effects of foods vs directly acting pharmacological agents like cocaine in order to refine concepts and models of “food addiction.”

## Supplementary Information

Below is the link to the electronic supplementary material.

Supplementary file1 (PDF 181 KB)

Supplementary file2 (PDF 157 KB)

## Data Availability

Data arising from this study will be made freely available upon request.
