# Supplementary material for: Cocaine-induced plasticity, motivation, and cue responsivity do not differ in obesity-prone vs obesity-resistant rats; implications for food addiction
Source: Psychopharmacology (Berl). 2023 Feb 18;240(4):853–70. doi: 10.1007/s00213-023-06327-5 (PMC10006066; doi:10.1007/s00213-023-06327-5)
Supplement: Supplementary file 1 — Supplementary file1 (PDF 181 KB) [file 213_2023_6327_MOESM1_ESM.pdf]

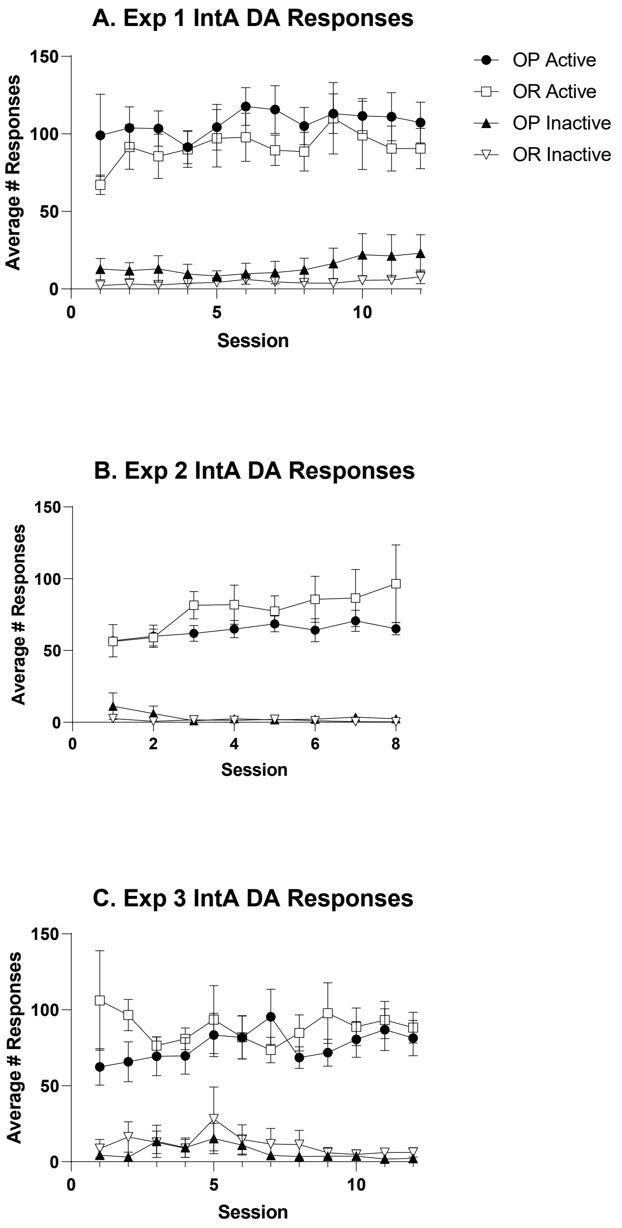

Supplemental Figure 1: Active vs inactive responses during the drug available (DA) period of IntA in experiments 1 (A), 2 (B), and 3 (C). Rats showed strong discrimination between the active and inactive ports, consistent with behavior during initial acquisition. Responses in the inactive port were low and stable throughout all sessions and were similar between OP and OR groups.
