# Supplementary material for: Cocaine-induced plasticity, motivation, and cue responsivity do not differ in obesity-prone vs obesity-resistant rats; implications for food addiction
Source: Psychopharmacology (Berl). 2023 Feb 18;240(4):853–70. doi: 10.1007/s00213-023-06327-5 (PMC10006066; doi:10.1007/s00213-023-06327-5)
Supplement: Supplementary file 2 — Supplementary file2 (PDF 157 KB) [file 213_2023_6327_MOESM2_ESM.pdf]

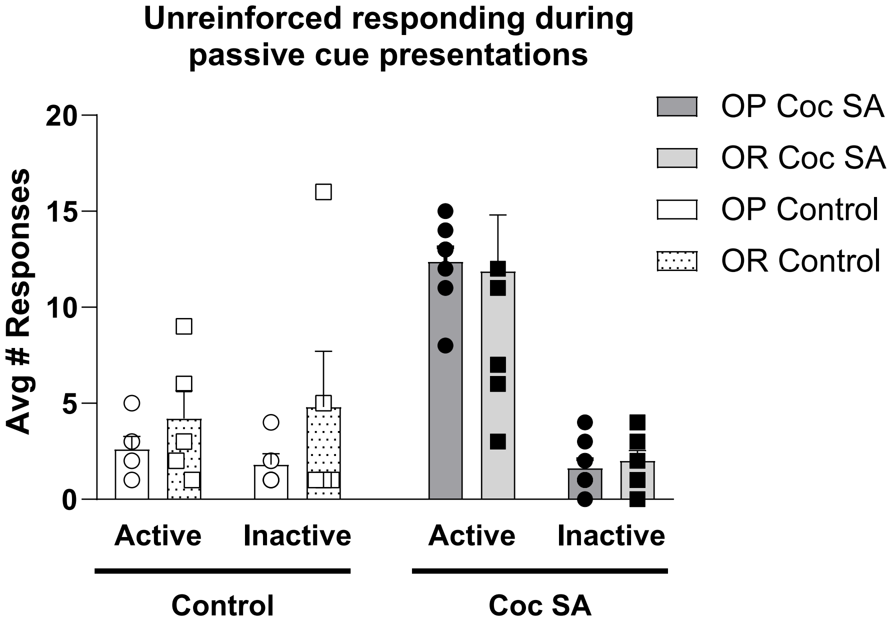

Supplemental Figure 2: Active and inactive responses during the c-Fos induction test. Note that responding in the active port during this test had no consequences. Rats with previous self-administration experience (Coc SA) responded more in the active than inactive nose-poke port (Two way RM ANOVA; main effect of active vs inactive; $F(1, 14) = 51.74$, $p<0.01$), while responding in controls with no prior self-administration experience was low and did not differ between the ports (Two way RM ANOVA; no main effect of active vs inactive; $F(1, 8) = 0.01$, $p = 0.92$). In addition, the magnitude of responding was similar between obesity-prone and obesity-resistant groups (OP, OR; Two way RM ANOVA; no main effect of strain; $F(1,22) = 0.26$, $p = 0.61$).
